# Osseointegration of porous titanium implants with and without electrochemically deposited DCPD coating in an ovine model

**DOI:** 10.1186/1749-799X-6-56

**Published:** 2011-11-03

**Authors:** Dong Chen, Nicky Bertollo, Abe Lau, Naoya Taki, Tomofumi Nishino, Hajime Mishima, Haruo Kawamura, William R Walsh

**Affiliations:** 1 Surgical & Orthopaedic Research Laboratories, Prince of Wales Hospital, University of New South Wales, Sydney, Australia; 2 Yokohama City University Medical Center, Yokohama, Japan; 3 University of Tsukuba, Tsukuba, Japan; 4 Ryugasaki Saiseikai Hospital, Ryugasaki, Japan

**Keywords:** Bone ingrowth, Interfacial shear strength, Calcium phosphate, Osteoconduction, Bone remodeling

## Abstract

**Background:**

Uncemented fixation of components in joint arthroplasty is achieved primarily through *de novo *bone formation at the bone-implant interface and establishment of a biological and mechanical interlock. In order to enhance bone-implant integration osteoconductive coatings and the methods of application thereof are continuously being developed and applied to highly porous and roughened implant substrates. In this study the effects of an electrochemically-deposited dicalcium phosphate dihydrate (DCPD) coating of a porous substrate on implant osseointegration was assessed using a standard uncemented implant fixation model in sheep.

**Methods:**

Plasma sprayed titanium implants with and without a DCPD coating were inserted into defects drilled into the cancellous and cortical sites of the femur and tibia. Cancellous implants were inserted in a press-fit scenario whilst cortical implants were inserted in a line-to-line fit. Specimens were retrieved at 1, 2, 4, 8 and 12 weeks postoperatively. Interfacial shear-strength of the cortical sites was assessed using a push-out test, whilst bone ingrowth, ongrowth and remodelling were investigated using histologic and histomorphometric endpoints.

**Results:**

DCPD coating significantly improved cancellous bone ingrowth at 4 weeks but had no significant effect on mechanical stability in cortical bone up to 12 weeks postoperatively. Whilst a significant reduction in cancellous bone ongrowth was observed from 4 to 12 weeks for the DCPD coating, no other statistically significant differences in ongrowth or ingrowth in either the cancellous or cortical sites were observed between TiPS and DCPD groups.

**Conclusion:**

The application of a DCPD coating to porous titanium substrates may improve the extent of cancellous bone ingrowth in the early postoperative phase following uncemented arthroplasty.

## Background

Uncemented fixation has been a major method employed in arthroplasty for decades [1,2]. To this end various rough and porous surfaces have been developed and applied in clinical use [3]. Aseptic loosening, however, is still a main cause of prosthesis failure [4]. In order to further improve bone-implant integration, highly porous or rough structures and surface coatings are continuously being investigated to enhance osteogenesis at the implant surface.

The recruitment and migration of osteogenic cells to the surface of implants to differentiate to osteoblasts forming new bone directly on the implant is referred to as contact osteogenesis [5,6]. Porous or rough surfaces can greatly increase surface area so as to attach large amount of surface adsorbing fibrins, which in turn cause increased numbers of osteo-differentiating cells to migrate to the bone-implant interface [5,6]. Plasma spraying is one of the most popular techniques used in the fabrication of porous surfaces for uncemented implantation [7,8]. It has been recognised that plasma spraying produces highly porous surfaces with open and interconnected pores, which can vastly improve bone ingrowth characteristics [7,9]. In addition, depending on porosity and the thickness of the porous coating, the compressive modulus of the porous substrate can be tailored to match that of cancellous bone, thus reducing the problems associated with stress shielding [7].

The osteoconductive nature of calcium phosphates can facilitate improved *de novo *bone formation at the bone-implant interface [10]. As such, they are often applied to implant substrates to improve bone-implant fixation [11,12]. Conventional hydroxyapatite (HA) coatings are also typically applied by a plasma spraying technique [13]. A limitation of this particular method is that HA may interfere with the structure, openness and interconnectivity of pores. An alternative method, electrochemical cathodic deposition, is performed in a solution containing dissolved calcium and phosphorus ions resulting in the deposition of a thin and uniform layer of calcium phosphate compound on the 3D porous substrate, with grain size ranging from the sub-micron scale to several micrometers [14]. Dicalcium phosphate dihydrate (DCPD) is one such osteoconductive coating which can be applied to a porous substrate by this method, without compromising pore openness and interconnectivity [15]. However, DCPD exists in living bone in a metastable phase [16], meaning that the length of time present *in vivo *is limited.

We conducted this study to determine whether an electrochemically-deposited DCPD coating could improve the extent of ingrowth and ongrowth for a highly porous titanium surface and whether the coating could enhance bone-implant interfacial shear strength. Our null hypothesis was that the DCPD coating would have no effect on interfacial cortical shear strength and osseointegration in either cortical or cancellous sites.

## Materials and methods

### Implants

One hundred and fifty plasma sprayed titanium implants (6 mm diameter, 22 mm long) without (TiPS group; n = 75) and with a DCPD coating (DCPD group; n = 75) were assessed in this study. The TiPS group served as the control, representing a medium used commonly in uncemented fixation. Pore size of the TiPS coating ranged from 50 to 200 μm with a microporosity of 35% and a thickness of 350 μm. The DCPD layer, applied using a process of electrochemical cathodic deposition exhibited an average thickness and dihydrate crystal size of 20 μm and 1-3 μm, respectively. Whilst not directly measured as part of this study it stands to reason that following the application of the DCPD coating effective pore size was in the order of 10 - 160 μm. Implants were manufactured by Aesculap AG, Germany.

### Experimental animal model

Twenty-one skeletally mature sheep (cross-bred Merino Wethers, 18 month-old, 54 ± 2 kg) were used in this study with ethical consent from our institutional Animal Care and Ethics Committee. Implants were inserted into cylindrical defects drilled bilaterally in the cancellous bone (n = 4 per animal) of the distal femur and proximal tibia and cortical bone (n = 2 per animal) of the tibial diaphysis. Sheep were sacrificed and specimens retrieved at five postoperative timepoints: 1 (n = 3), 2 (n = 3), 4 (n = 6), 8 (n = 3) and 12 (n = 6) weeks. Three sheep per time point provided a total of 6 cortical and 12 cancellous specimens per group at each timepoint. Three additional animals were allocated to the 4 and 12 week groups to ensure a sufficient sample size and statistical power to detect a significant difference in interfacial shear strength. In these animals an additional 4 cortical implants were inserted as described below. These timepoints were chosen based on our previous publications with this animal model [10,17,18].

The bilateral surgical implantation model used in this study has previously been described in detail [10,17,18]. For cancellous implantation, a 4 cm longitudinal incision was made from the medial epicondyle across the knee joint line to a point approximately 2 cm below medial tibial plateau. The medial femoral condyle and the medial tibial plateau were exposed. The implantation centre in the femur was positioned approximately 1 cm anterior and 1 cm inferior to the medial epicondyle, with the axis of the drilled defect being perpendicular to the surface of medial femoral condyle. The implantation point in tibial plateau was midway along the anteroposterior dimension of the tibial plateau and 8 mm distal to the proximal tibial joint surface. A 5 mm diameter hole was first drilled in cancellous bone which was then over-drilled to a 5.5 mm diameter. The 6.0 mm diameter implant was inserted in a press fit manner using a custom-made impactor.

For cortical implantation a second incision was made to expose the tibial diaphysis. Three bicortical holes were created using 5 mm and 6 mm diameter drills in sequence. Holes in the tibial shaft were spaced approximately 2 cm apart in an effort to avoid stress concentrations and decrease the likelihood of fracture. Cortical implants were inserted in a line-to-line fashion.

Sheep were free to mobilize in their pen and fully weight-bear. Implants were retrieved at harvest and processed for mechanical, histologic and histomorphometric endpoints.

### Mechanical testing

Mechanical testing was conducted to evaluate interfacial shear strength of cortical bone samples as previously described [10,17,18]. Implants were displaced at a constant rate (5 mm.min^-1^) using an 858 Bionix Servohydraulic Materials Testing Machine (MTS Systems Inc., MN, USA). Peak pushout force (N), stiffness (N/mm) and energy-to-failure (J) were determined from load-displacement output using Matlab (Matlab R2009a, MathWorks Inc. MA, USA). Interfacial shear-strength (MPa) values were derived from the combination of peak pushout force and mean cortical thickness (mm) determined from the PMMA embedded sections (as described below).

### Histology

Retrieved cancellous and mechanically-tested cortical bone specimens were fixed in 10% phosphate buffered formalin, subsequently dehydrated in increasing concentrations of alcohol (70 - 100%) and embedded in polymethyl methacrylate (PMMA) for histological and histomorphometric assessment. Two sections were cut from each embedded cancellous specimen and one from each cortical specimen using a Buehler Isomet Saw (Buehler, IL, USA). For the cancellous samples, multiple sections were taken perpendicular to the long axis of the implant, whilst for cortical samples the single section was coincident with the implant long axis. Sections were ground, polished and sputter-coated in gold (25 nm thickness) using an Emitech K550× Gold Sputter Coater (Quorum Technologies Ltd, Ashford, UK), followed by imaging with back scattered electron microscopy (BSEM) imaging on a HITACHI S-3400 SEM (Hitachi High-Technologies Corporation, Tokyo, Japan). Low power overviews of the cortical specimens were used to obtain values for cortical thickness in the derivation of interfacial shear strength.

Following analysis by SEM a 30 μm thick section was cut from each embedded specimen using a Leica SP1600 saw microtome (Leica Microsystems, Nussloch, Germany) and stained with methylene blue and basic fuchsin and observed under a light microscope.

### Histomorphometry

Percentage bone ingrowth was calculated based on SEM images using Bioquant Nova Prime image analysis software (BIOQUANT Image Analysis Corporation, TN, USA). Both cancellous and cortical specimens were analysed using similar techniques. The porous coating region of the specimen, new bone and void, was selected using a rectangular region of interest (ROI). Bone ingrowth fraction was calculated as bone volume divided by available void (i.e. total pixel area minus the pixels occupied by titanium). In this was bone ingrowth was normalised to the amount of available void. Bone ongrowth rate was calculated on SEM images using Matlab. Percentage bone ongrowth was also determined, defined as bone contact area divided by implant perimeter in each ROI.

### Statistical analysis

Mechanical and histomorphometric data were analysed with SPSS 17.0 software (SPSS Inc., IL, USA). Data were analysed using an ANOVA with Tukey's *post hoc *testing. Statistical significance was considered where *P *< 0.05.

## Results

### Bone-implant interface mechanical properties

Interfacial shear-strength data is summarised in Table 1. No significant difference in interfacial shear-strength, stiffness and energy-to-failure between the DCPD and TiPS groups at each timepoint was found (*P *> 0.05). The DCPD coating had no effect on implant fixation in the cortical sites up to 12 weeks postoperatively. Interfacial shear-strength increased significantly with time for both implant types (*P *< 0.05). For the DCPD group, shear-strength increased after 2 weeks and the differences were significant between 4 and 8 weeks, 4 and 12 weeks, as well as 2 and 8 weeks (*P*-values of 0.036, 0.001 and 0.005, respectively). For the TiPS group, interfacial shear strength also increased with time, with the increase being significant between 4 and 12 weeks as well as 2 and 8 weeks (*P*-values of 0.001 and 0.024, respectively).

**Table 1:** Interfacial shear strength results for the DCPD and TiPS implant groups as a function of postoperative timepoint.

Time (weeks)	Shear Strength (MPa)		
	
	DCPD	TiPS	P value
**1**	2.38 (1.81)	0.11 (0.02)	0.999

**2**	2.15 (2.64)	2.29 (2.02)	0.999

**4**	10.61 (4.35)	16.99 (11.34)	0.608

**8**	24.88 (4.35)	22.29 (6.09)	0.999

**12**	28.32 (5.43)	29.06 (8.22)	0.999

(Mean ± SD)

**Figure 1:**
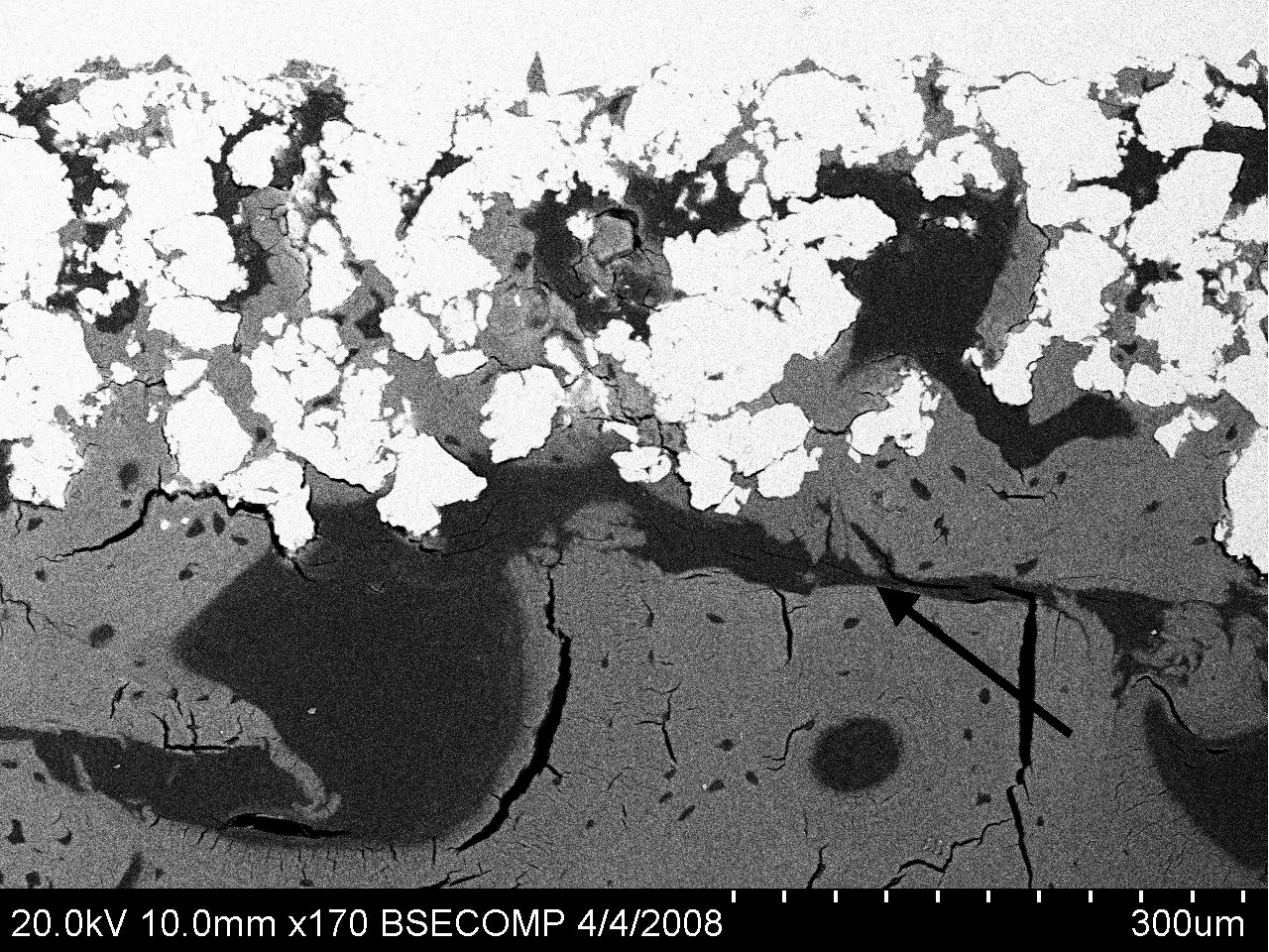
**SEM image of an implant from the DCPD group depicting the failure location (black arrow) after push-out testing**.

The mode of failure for the plasma sprayed titanium implants is illustrated in Figure 1, where the fracture plane was typically coincident with the host bone/de novo bone interface. An exception to this rule were the 1 and 2 week timepoints, where the fracture plane was coincident with the de novo bone implant interface, and which may be indicative of insufficient appositional bone growth. For all mechanical testing samples, regardless of timepoint, no damage to or delamination of the porous titanium domain was observed, despite mean ultimate interfacial shear strength values 12 weeks postoperatively of 28.3 ± 5.43 MPa and 29.06 ± 8.22 MPa for the DCPD and TiPS groups, respectively.

### Bone ongrowth

**Figure 2:**
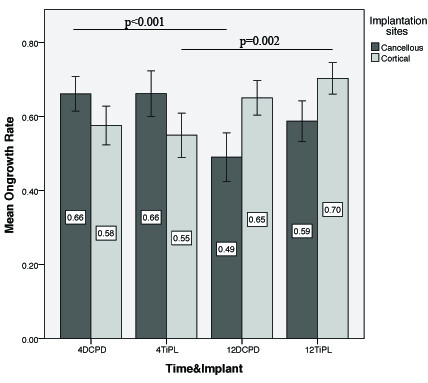
**Mean percentage bone ongrowth for TiPS and DCPD groups as a function of implantation site and time**. Note that implant group and timepoint are combined in the x-axis categorical variable (Mean ± SE).

No significant differences in ongrowth were found between DCPD and TiPS groups in either the cancellous or cortical implantation sites (*P *> 0.05) (Figure 2). Mean ongrowth in the cancellous site decreased from 4 to 12 weeks in both groups, where this reduction was significant for the DCPD coating (*P *< 0.001) only. On the contrary, mean cortical bone ongrowth increased from 4 to 12 weeks for both groups, where this increase was significant for the TiPS coating (*P *= 0.002). Mean percentage bone ongrowth for the cortical implantation sites appeared lower than cancellous site at 4 weeks in both DCPD and TiPS groups, although the difference was not significant. However, cortical bone ongrowth rate surpassed cancellous ongrowth rate in both groups at 12 weeks, which was significant for the DCPD coating (*P *= 0.001).

### Bone ingrowth

**Figure 3:**
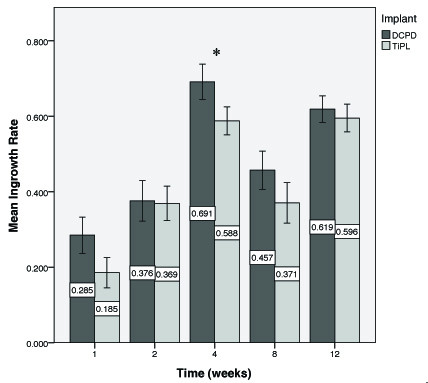
**Mean percentage ingrowth for both implant groups in cancellous bone as a function of time**. * denotes P = 0.003. (Mean ± SE).

Mean percentage bone ingrowth for the DCPD and TiPS groups in the cancellous sites ranged from 29% to 69% and 18% to 60%, respectively (Figure 3). DCPD implants showed higher mean percentage bone ingrowth at all time points, with the difference being significant at 4 weeks (*P *= 0.003) only. In the cortical sites no significant difference in bone ingrowth rate was observed between DCPD and TiPS at either timepoint (*P *> 0.05).

Mean bone ingrowth was generally higher in cancellous bone than cortical bone for both TiPS and DCPD groups at 4 weeks, although the differences were not significant (*P *> 0.05). In contrast, cortical sites generally exhibited higher bone ingrowth rate than cancellous site at 12 weeks, which was significant for the TiPS coating (*P *< 0.001).

### Histological findings

**Figure 4:**
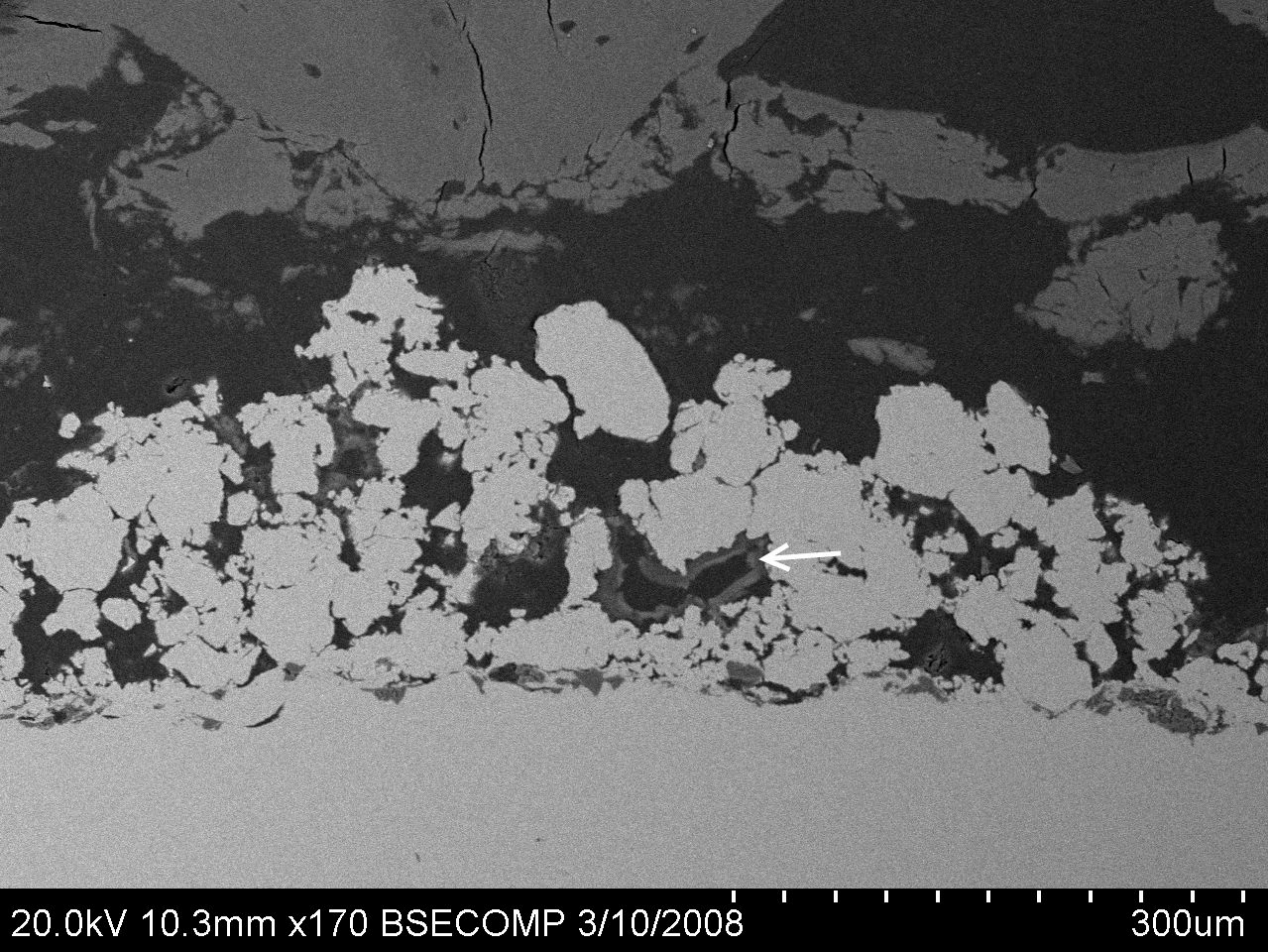
**Traces of DCPD were visible from DCPD sections at 1 week**.

At 1 week following surgery, bone debris still could be seen around both TiPS and DCPD implants, indicating it had yet to be fully resorbed. Only traces of DCPD coating were visible from the BSEM images (Figure 4), suggesting substantial resorption of DCPD coating had taken place following 1 week *in situ*.

**Figure 5:**
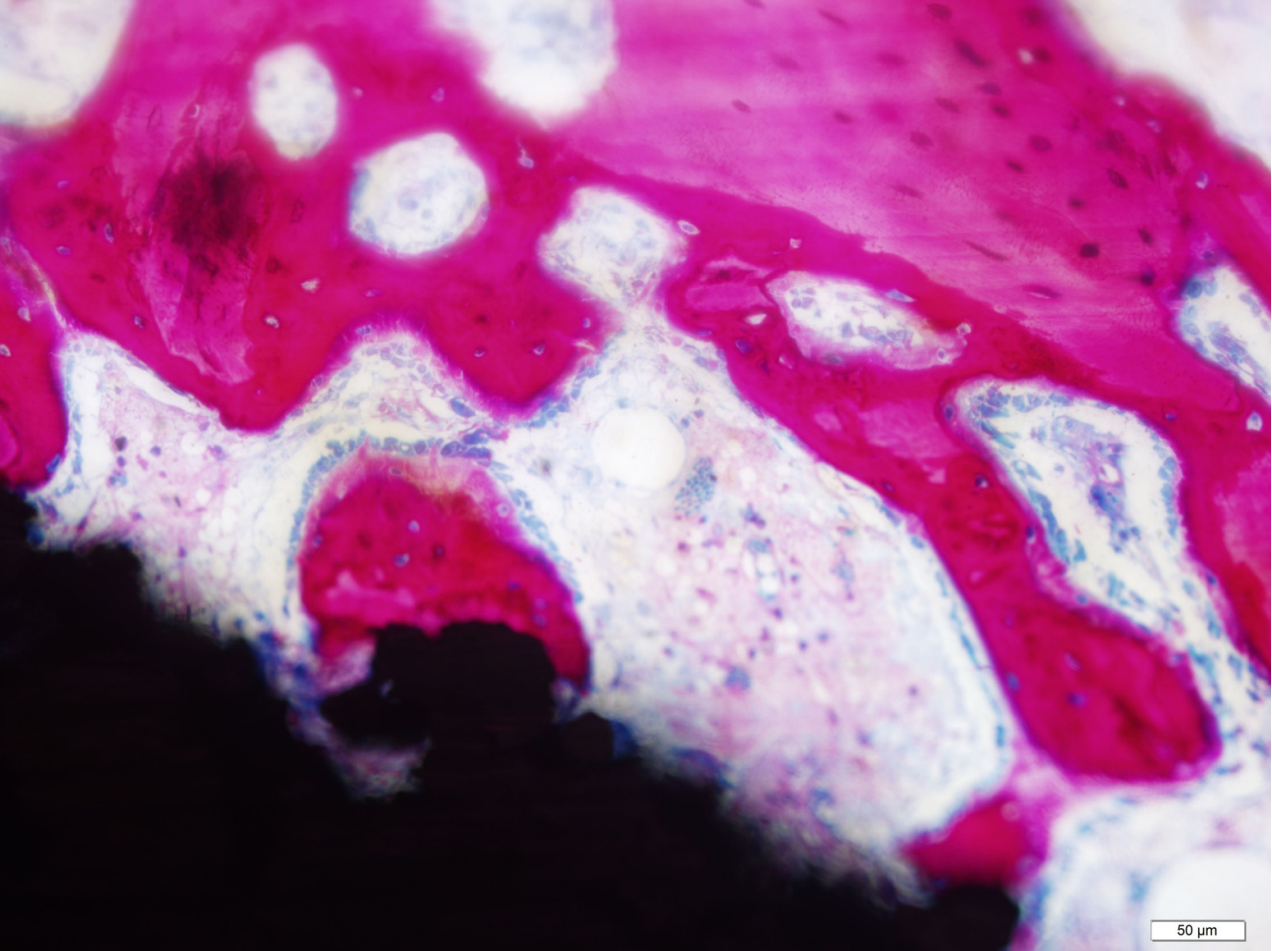
**Osteoblasts were enlarged, roundish and in layers on newly formed bones directly on porous implant surface and on opposite surrounding bone**. Image taken 2 weeks postoperatively.

**Figure 6:**
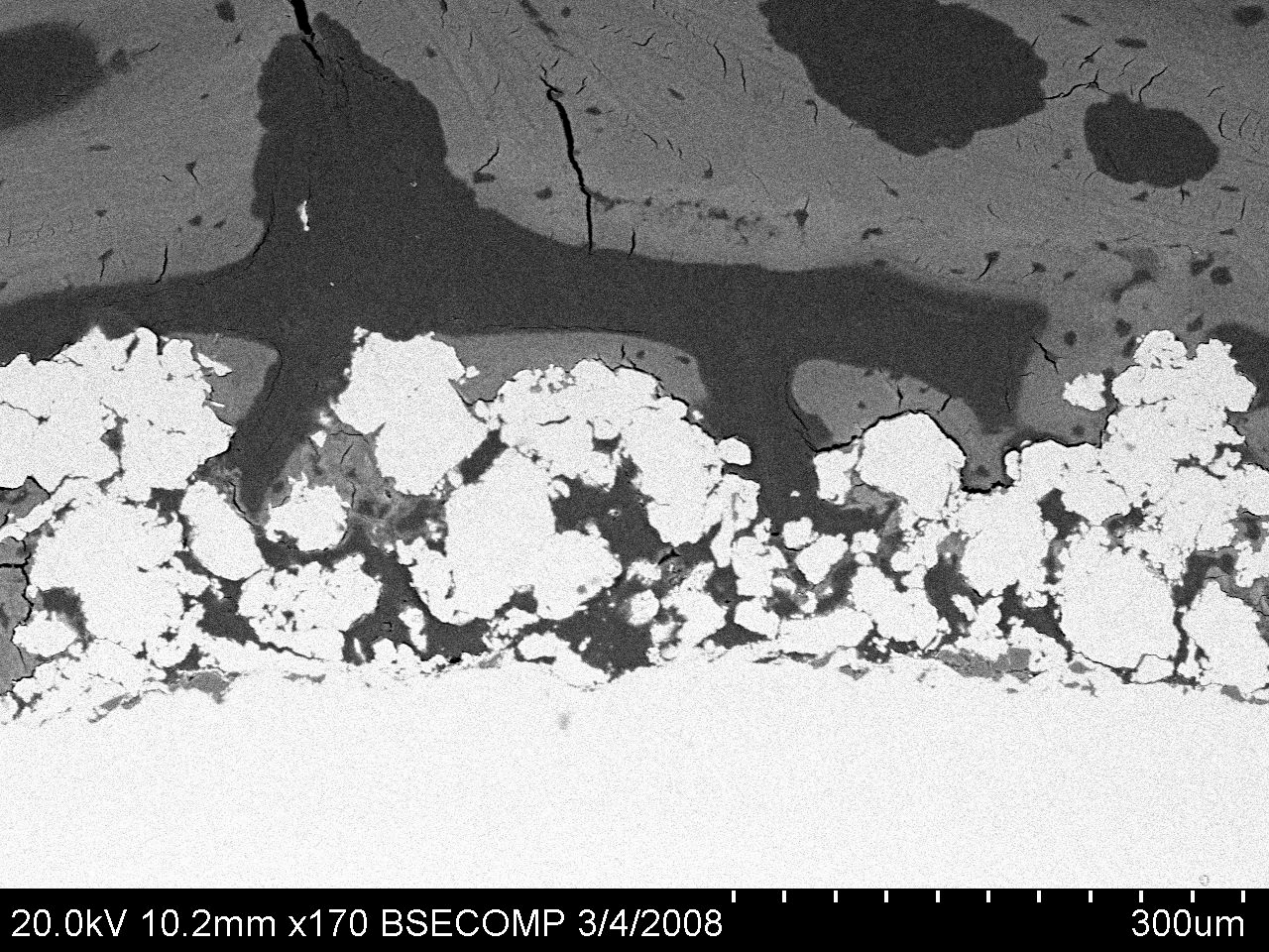
**SEM image depicting de novo bone formation on and extending to within the porous surface at 2 weeks postoperatively**.

Analysis of TiPS and DCPD implants at 2 weeks illustrated the initial de novo woven bone formation and resorption of bone debris. The new bone appeared as a deep red colour in the histology images, indicating newly formed bone growing directly on the implant surface (Figure 5). Osteoblast lines could be seen on newly formed bone directly on the porous implant surface. The osteoblasts appeared enlarged, roundish and in layers, indicating contact osteogenesis had been active at 2 weeks. They could also be seen on nearby new bone, suggesting distance osteogenesis. New bone could also be observed growing deep into the pores, extending to the cylindrical implant substrate (Figure 6). Both osteogenic mechanisms were evident in the TiPS and DCPD specimens. There was no evidence of residual DCPD coating at 2 weeks post-implantation.

**Figure 7:**
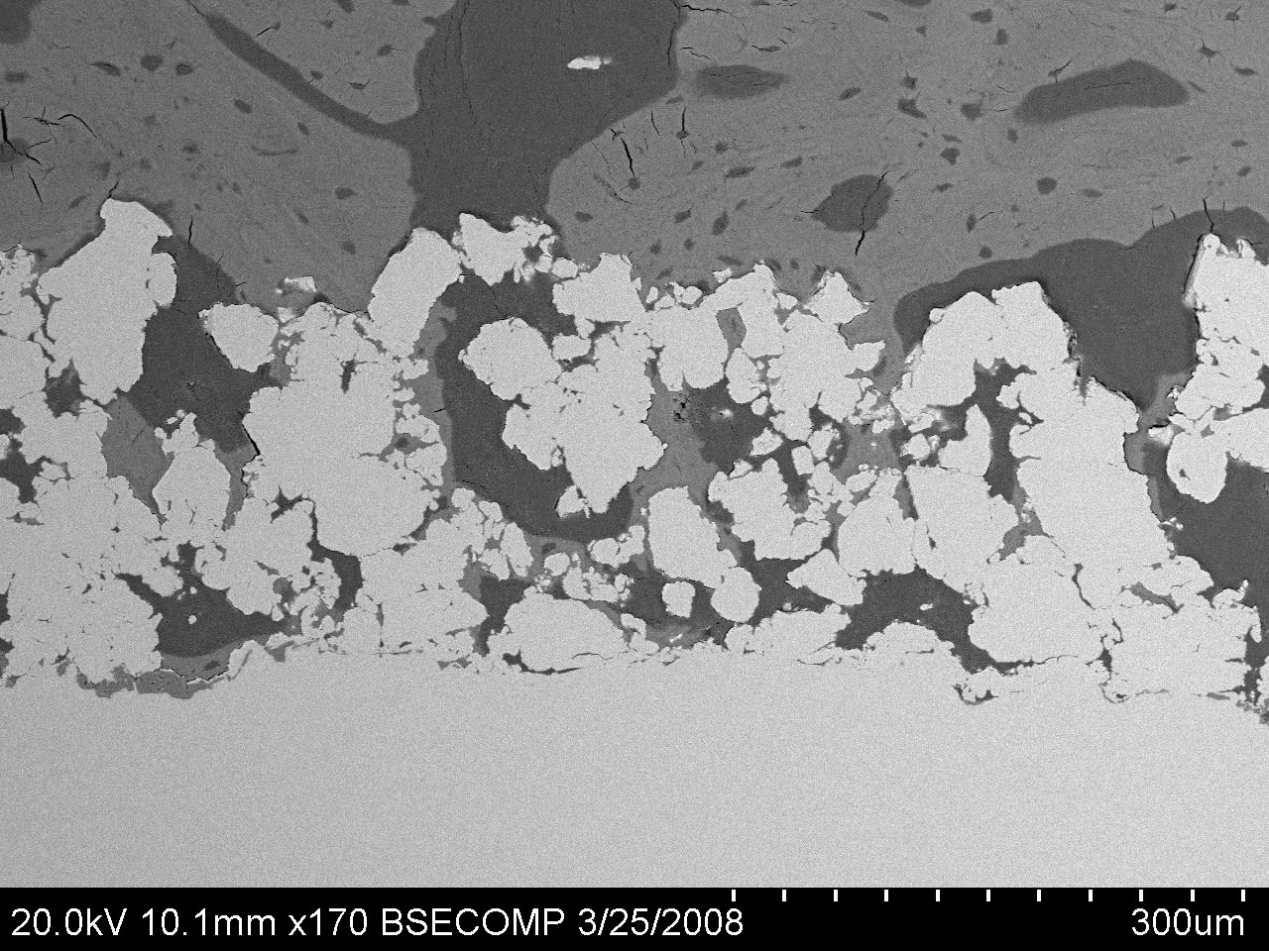
**SEM image depicting a continuation of the trabecular structure of the cancellous bone to within the porous implant domain, despite the barrier provided by the coating itself**. In this image bone can be seen growing onto the cylindrical implant substrate.

**Figure 8:**
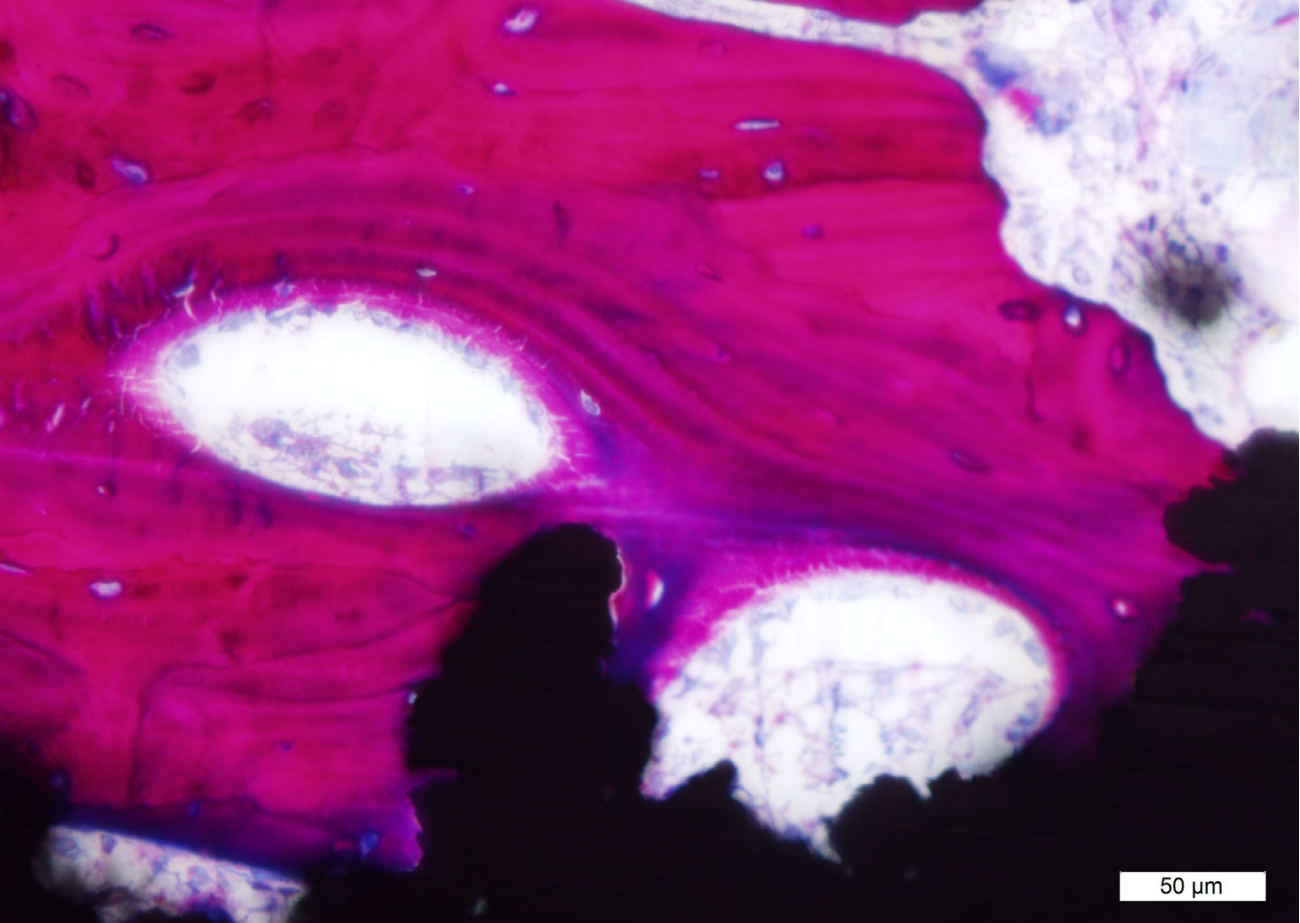
**Haversian canals and lamellar bone, indicative of mature bone were clearly seen at 12 weeks**.

Images of both the TiPS and DCPD mediums at 4 weeks illustrated that the newly deposited bone resembled normal trabeculae, growing from outside to inside pores and exhibiting continuous curves, despite the intervening presence of the titanium pore walls (Figure 7). Haversian canals were occasionally seen in the images at 4 and 8 weeks, indicating remodelling. At 12 weeks, mature Haversian canals could be seen in both TiPS and DCPD implants. Osteocytes were more evenly distributed and lamellar bone could be clearly identified (Figure 8).

## Discussion

Electrochemical cathodic deposition is a method employed to apply a thin and uniform layer of calcium phosphate coating on a porous implant surface. Metallic implants are submerged in an electrolyte bath containing dissolved calcium and phosphorus ions and connected to an external power supply [14]. A thin DCPD layer with grain size ranging from 1-3 μm is deposited on and within the porous implant surface, without compromising pore openness and interconnectivity [15]. DCPD dissolution is mainly affected by volume diffusion [19]. In this study the DCPD layer was found to be mostly dissolved at 1 week, with only trace amounts present at 2 weeks, which is consistent with other reports in the literature [20,21].

DCPD is believed to act as a heterogeneous centre for HA growth in early bone formation [22]. For this reason it has been postulated that the thin calcium phosphate coating will improve bone ongrowth and ingrowth of porous implant surfaces to achieve rapid and early bone-implant interface integration and stability. Our results suggest that a DCPD coating has the potential to improve the extent of cancellous bone ingrowth in the early postoperative period (Figure 3). This finding is consistent with an *in vitro *study showing higher cell attachment ability on calcium phosphate compound samples in the early stages [23]. Simank et al [15] detected no significant difference in the mechanical fixation or bone formation throughout porous titanium implants coated with either an osteoinductive growth and differentiation factor-5 (GDF-5) or osteoconductive DCPD coating [15]. The mean bone ingrowth rate in the DCPD group was approximately 66% in cortical bone at 4 weeks, which compares well with values of 60% and 48% previously reported for a porous beaded coating with and without a 50 μm HA coating at 4 weeks in an ovine model [10].

In this study the cancellous implantation sites presented with higher mean bone ingrowth and ongrowth values than in the cortical bone sites at 4 weeks postoperatively for both DCPD and TiPS groups. Whilst this mean increase was not statistically distinguishable this finding is consistent with the knowledge that cancellous bone heals at a faster rate than cortical bone [24]. On the other hand, bone ingrowth and ongrowth in cortical bone sites showed generally higher percentage values than in cancellous bone at 12 weeks for both groups. Ostensibly, this result at 12 weeks is indicative of the compact nature of cortical bone. In joint arthroplasty the primary mode of fixation for uncemented tibial trays, femoral components and acetabular cups is indeed via cancellous bone ongrowth and ingrowth. Possible effects which the differential in ongrowth and ingrowth patterns observed in this study may have on uncemented fixation of joint components remains unknown.

Another striking feature in the current study was the seeming preservation of trabecular bone structure to within the porous coating domain (Figure 7), despite the presence of intervening titanium. Because trabecular bone tends to adapt to direction of mechanical stress [25,26] this phenomenon may indicate that mechanical loads were indeed transmitted through the thin titanium pore walls. This observation supports the potential of selective manufacturing to limit the effects of stress-shielding by tailoring the elastic modulus of mediums for hard tissue infiltration. Ryan and colleagues [7] have demonstrated that the compressive modulus of porous metals is better matched to cancellous bone as compared to solid metals. This phenomenon of the continuation of trabecular bone architecture to within the porous coating has not previously been observed for thick-walled porous surfaces, such as beaded constructs [18].

An implant exhibiting an osteoconductive coating can stimulate new bone growth directly on the implant surface [8,9] and improve uncemented prosthesis fixation in the early postoperative period [27,28]. In this study, the plasma sprayed titanium porous surface both with and without the electrochemically-deposited DCPD coating exhibited de novo bone formation on the implant surface as early as two weeks after implantation (Figure 5 and Figure 6). At this timepoint osteoblasts were seen lining new bone on both the implant surface and adjacent host bone (Figure 5). Contact osteogenesis in both DCPD and TiPS groups was in agreement with a report that porous concave coatings can stimulate osteogenic cells differentiating to osteoblasts [29].

The percentage ingrowth for both test materials in the current study averaged approximately 37% at 2 weeks, as compared to the 13% ingrowth obtained for a porous tantalum implant [30]. Tantalum has been recognized as having excellent bone and fibrous ingrowth properties, allowing for rapid and substantial bone and soft tissue attachment [31]. Direct comparison of these results is fraught with difficulty, though, due to differences between studies in terms of implant parameters (porosity and coating thickness), implantation site and species. Regardless, the results of the current study support the osteoconductive potential of a highly porous titanium surface with a DCPD coating.

Evidence of remodeling in the cancellous sites was observed in both DCPD and TiPS groups as early as 4 and 8 weeks, with Haversian canals identified at 12 weeks (Figure 5). In addition, considerable amounts of lamellar bone and evenly distributed osteocytes were clearly seen in surrounding bone on both DCPD and TiPS sections at 12 weeks. The rate of remodeling in the current study is in contrast to other previous uncemented implant fixation studies in sheep [32,33] where woven bone and lamellar matrix persisted three months postoperatively. This remodeling rate may be attributed to the highly porous surface and the press-fit insertion manner adopted in current study.

Mechanical testing revealed no difference between DCPD and TiPS at either timepoint. When selecting a soluble material for coatings, the match of resorption rate and bone regeneration rate must be taken into account. If resorption rate is faster than regeneration, there may be a void left by the absorbed material, which can potentially compromise bone and implant contact [13]. The shear strength of DCPD group was not lower than the control group in the current study, although the DCPD coating appeared mostly absorbed at 1 week and almost completely at 2 weeks. The mechanical similarity between DCPD and TiPS group in the two early time points indicated the thin (20 μm) and highly soluble DCPD coating will not compromise bone-implant interface mechanical stability in early stage.

The failure mode for both implant types from 4 to 12 weeks postoperatively was primarily at the interface between de novo formed and host bone. The failure mode illustrated that shear strength depends on the amount and strength of surrounding new bone, which can also be correlated to a study showing that mechanical stability of rough titanium implants depends on the amount of bony tissue surrounding the implant [15]. This may be the reason why higher ingrowth did not result in higher shear strength in DCPD implants. The increase of mechanical strength with increasing time may be due to the increasing amounts of mature surrounding bone.

## Conclusion

The study of plasma sprayed porous titanium surface coated with and without DCPD demonstrated electrochemically deposited thin layer of DCPD with fine grain size can improve bone ingrowth *in vivo*. Mechanical results indicate that the thin and soluble DCPD had neither a positive nor negative effect on interfacial shear strength and implant stability in cortical bone. Moreover, analysis of the failure mode suggests that the bone bonding strength of the porous surface depends on the amount and maturity of surrounding new bone for both groups. As expected, an improvement in interfacial shear strength for both implant types with time was observed, continuous with the mechanical advantage of bony remodeling.

Cancellous bone implantation was associated with higher bone ingrowth and ongrowth at the early stage, whilst cortical bone implantation had more bone ingrowth and ongrowth than cancellous bone at 12 weeks. The continuity of trabecular bone to within the porous coatings (Figure 7) also indicates the adaptation of the highly porous surface structure to cancellous bone. The implantation of the porous surface implants by press-fit insertion demonstrated excellent early new bone formation and remodelling.

Finally, electrochemical deposition has the potential to produce calcium phosphate compounds with sub-micron sized grains which may lead to higher cell adhesion and osteoblast activity [34]. The effect of such coatings may be examined in the future.

## Competing interests

Funds for this study were received by our Institution from BBraun Aesculap Japan. Co. No author of this paper was a direct beneficiary of such funding.

## Authors' contributions

WRW is credited with both conception and design of the study. DC performed the animal surgery and, along with WRW, AL and NB was also involved with and responsible for the processing of data, statistical analysis and interpretation of results. All authors contributed equally to drafting and critical review of the manuscript.
